# Glaucoma: the retina and beyond

**DOI:** 10.1007/s00401-016-1609-2

**Published:** 2016-08-20

**Authors:** Benjamin Michael Davis, Laura Crawley, Milena Pahlitzsch, Fatimah Javaid, Maria Francesca Cordeiro

**Affiliations:** 1 UCL Institute of Ophthalmology, 11-43 Bath Street, London, UK; 2 Western Eye Hospital, Imperial College Healthcare NHS Trust, 153-173 Marylebone Road, London, UK

**Keywords:** Glaucoma, Retina, Neurodegeneration, Cell death, Imaging, Retinal ganglion cell

## Abstract

Over 60 million people worldwide are diagnosed with glaucomatous optic neuropathy, which is estimated to be responsible for 8.4 million cases of irreversible blindness globally. Glaucoma is associated with characteristic damage to the optic nerve and patterns of visual field loss which principally involves the loss of retinal ganglion cells (RGCs). At present, intraocular pressure (IOP) presents the only modifiable risk factor for glaucoma, although RGC and vision loss can continue in patients despite well-controlled IOP. This, coupled with the present inability to diagnose glaucoma until relatively late in the disease process, has led to intense investigations towards the development of novel techniques for the early diagnosis of disease. This review outlines our current understanding of the potential mechanisms underlying RGC and axonal loss in glaucoma. Similarities between glaucoma and other neurodegenerative diseases of the central nervous system are drawn before an overview of recent developments in techniques for monitoring RGC health is provided, including recent progress towards the development of RGC specific contrast agents. The review concludes by discussing techniques to assess glaucomatous changes in the brain using MRI and the clinical relevance of glaucomatous-associated changes in the visual centres of the brain.

## Introduction

Glaucoma is a leading cause of irreversible blindness worldwide and is associated with characteristic damage to the optic nerve and patterns of visual field loss due to retinal ganglion cell (RGC) degeneration. Intraocular pressure (IOP) is regarded as the major risk factor. There are estimated to be over 60 million people worldwide with glaucomatous optic neuropathy of which 8.4 million are blind [172]. The global incidence of glaucoma is anticipated to increase to 76 million by 2020 and 111.8 million by 2040 [172]. Glaucoma is an umbrella term encompassing various subtypes of the condition, but the structural damage to the optic nerve is common throughout. The formal definition of clinical glaucoma as suggested by Casson et al. is [22]:

*“a group of ocular disorders of multifactorial aetiology united by a clinically characteristic optic neuropathy with potentially progressive, clinically visible changes at the optic nerve head (ONH), comprising focal or generalized thinning of the neuroretinal rim with excavation and enlargement of the optic cup, representing neurodegeneration of retinal ganglion cell axons and deformation of the lamina cribrosa; corresponding diffuse and localized nerve*-*fibre*-*bundle pattern visual field loss may not be detectable in early stages; while visual acuity is initially spared, progression can lead to complete loss of vision; the constellation of clinical features is diagnostic”*

Glaucoma is principally divided into open-angle or angle-closure subtypes, where angle closure refers to the presence of appositional or synechial iridotrabecular contact resulting in the obstruction of the trabecular meshwork and elevated IOP [46]. Both open-angle glaucoma and angle-closure glaucoma can be further subdivided into primary or secondary diseases, where primary refers to the observation of characteristic optic neuropathy in the presence of normal or elevated IOP with no distinguishable pathological cause. Secondary disease refers to an elevated IOP with an identifiable pathological cause, such as inflammation, trauma, neovascularisation, pigment dispersion, and pseudoexfoliation [22]. Finally, primary open-angle glaucoma (POAG) can be arbitrarily further subdivided depending on the IOP status of the patient. Normal tension glaucoma (NTG) was a commonly used term that has fallen out of favour recently. NTG refers to POAG, where patients exhibit a normal IOP (IOP typically <97.5 percentile of population, <21 mmHg) versus POAG with an elevated IOP (IOP typically >97.5 percentile of population), although this distinction is arbitrary and may not be clinically relevant [57]. It is also important to note that not all high IOP causes damage to the optic nerve, a condition known as ocular hypertension [93]. This reinforces the theory that although IOP is currently the only modifiable risk factor in glaucoma, there are other factors influencing the progression of RGC loss. IOP reduction, even if the presenting IOP is normal, is still the principle way of managing this condition and is very effective with a 20 % reduction in pressure, reducing the risk of progression by 50 % [93].

A recent systematic review and meta-analysis by Tham et al. estimates a global glaucoma prevalence of 3.54 % of the population aged between 40 and 80 years of age [172]. In this study, the prevalence of glaucoma subtypes was found to vary by region and ethnicity, with the incidence of POAG varying between 4.20 % (Africa) and 2.31 % (Asia), and primary angle closure glaucoma varying between 1.09 % (Asia) and 0.26 % (North America). Additional risk factors for POAG identified using Bayesian Meta-Regression Modelling include: age, gender, and environment. A summary of the proposed POAG risk factors is presented in Table 1.

**Table 1:** Overview of identified risk factors for POAG

Parameter	Trend	Key references
IOP elevation	Increased incidence and prevalence. Increased rate of progression (RoP); elevated IOP compared to normal IOP—Canadian glaucoma study odds ratio (OR) 1.19, European glaucoma prevention study OR 1.12	Miglior et al. (2007) Chauhan et al. (2008) Gordon et al. (2002)
Age	Increased incidence and prevalence; Hispanics and people of European ancestry showed a steeper increase in POAG prevalence with age compared with African and Asian populations	Leske et al. (2008) Friedman et al. (2007)
Race	Increased incidence and prevalence of POAG in African (highest prevalence worldwide, 4.2 % of African population), Afro-Caribbean and coloured American population	Friedman et al. (2007)
Inheritance	Increased incidence and prevalence First degree relative with glaucoma increases POAG risk ten-fold POAG exhibits minor but significant heritability (twin study)	Leske et al. (2008) Le et al. (2003) Gordon et al. (2002) Wolfs et al. (1998)
Gender	Increased incidence and prevalence in men (OR 1.36)	Tham et al. (2014)
Living area	Higher incidence for people living in urban habitation areas than those in rural areas (OR 1.58)	
Myopia	Increased RoP, OR of 1.65 between low myopia (myopia up to -3D) and glaucoma and OR of 2.46 between high myopia (≤-3D myopic) and glaucoma	Marcus et al. (2011)
Cornea thickness	Increased incidence and prevalence in patients presenting with a thin cornea thickness, African American participants had a thinner central corneal measurement (554.9 ± 38.5 µm) than other participants (578.3 ± 36.5 µm)	Leske et al. (2008) Gordon et al. (2002)
Pseudoexfoliation syndrome (PES)	Increased incidence and prevalence Strong risk factor for POAG progression Varies by ethnicity and geographic location (high prevalence in Scandinavian populations)—patients with bilateral PES tend to be older and have a higher incidence of glaucoma or ocular hypertension when compared to patients with unilateral involvement Increased incidence and prevalence in men and patients between 52–64 (0.6 %) and 75–85 (5 %) years old Increased RoP due to higher fluctuations in IOP levels and reduced response to topical therapy	Le et al. (2003) Kozart et al. (1982) Leibowitz et al. (1980)
Cup–disc ratio	Increased incidence in larger vertical cup–disc ratio > 0.7 and larger horizontal cup–disc ratio, cup-disc ratio has to be corrected for disc size- highest diagnostic power compared to other optic disc parameters for separating healthy subjects from preperimetric glaucoma patients varies with race-African Americans had a larger vertical cup–disc ratio (0.45 ± 0.18) compared with other participants (0.37 ± 0.20)	Gordon et al. (2002) Le et al. (2003)
Blood pressure abnormality	Increased incidence and prevalence with low systolic pressure, increased severity with diurnal pressure fluctuations and lower diastolic blood pressure, especially during night time Increased RoP in patients taking hypertensive medication in the evening—evening rhythm is associated with significantly lower perfusion pressures at night	Leske et al. (2008)
Obstructive sleep apnoea syndrome (OSAS)	Increased incidence and prevalence (odds ratio 1.41–1.96)	Shi et al. (2015)
Diabetes	Diabetes patients showed a higher prevalence than healthy patients (odds ratio 1.48), risk of glaucoma increased by 5 % for each year since diagnosis	Zhao et al. (2015) Zhou et al. (2014)
Medication	Increased RoP and incidence when using systemic calcium channel blockers (1.8 fold higher risk)	Müskens et al. (2007)
Migraine	Higher incidence and prevalence in normal tension glaucoma (21.4 %) compared to POAG (13.1 %) and PACG (10.1 %)	Gordon et al. (2002) Gramer et al. (2015)
Cerebrovascular accident	Increased RoP, Blue mountain eye study—glaucoma patients had a non-significant increased risk of cardiovascular death (relative risk RR 1.46), increased cardiovascular mortality in glaucoma patients <75 years (RR 2.78)	Gordon et al. (2002) Lee et al. (2006)
Metabolic syndrome (includes obesity, smoking, alcohol intake)	Increased severity in patients with obesity showing excessive orbital fat and increased blood viscosity which may increase episcleral venous pressure and reduce aqueous outflow Increased RoP in women showing a low body mass index—suggested to be a result of the relationship between BMI and cerebral spinal fluid pressure	Mori et al. (2000) Ramdas et al. (2011) Zeevaart et al. (2009) Berdahl et al. (2012)
Age-related macular degeneration	Increased prevalence in 4–40 % of glaucoma patients in predominantly Caucasian populations	Le et al. (2003) Peräsalo et al. (1994) Tarkkanen et al. (2008)

The diagnosis of glaucoma is presently achieved using a combination of both functional and structural assessments. The standard automated perimetry is primarily used to assess functional deficits in glaucoma patients, while structural diagnostic tools are principally based on the quantification of retinal nerve fibre layer (RNFL) thickness changes by optical coherence tomography (OCT) and disc tomography (confocal laser-scanning tomography) to assess structural changes at the site of the optic nerve head (ONH). Clinical assessment of the optic disc using slit lamp examination is not surpassed by recent advances in structural imaging techniques. Slit lamp biomicroscopy is required to assess the colour or pallor of the disc, disc haemorrhages, and factors associated with secondary glaucomas, such as neovascularisation of the disc, retina, iris and drainage angle, uveitis, pseudoexfoliation, pigment dispersion, primary or secondary iris transillumination, trauma, and gonioscopic assessment of the drainage angle.

IOP, presently the only modifiable risk factor for glaucoma, is assessed using tonometry devices. The gold standard is Goldman applanation tonometry (GAT), but other devices exist and are used in optometric practice and in research settings, such as non-contact tonometry (e.g. Pulsair), rebound tonometry, and ocular pulse analyser [127]. An increasingly recognised limitation of IOP measurement by tonometry is that a thicker central cornea, as measured by ultrasound pachymetry, leads to a higher measured IOP than is present, whereas a thinner central cornea underestimates the true IOP [107].

A major drawback of current approaches to glaucoma diagnosis includes the inability to positively diagnose glaucoma before considerable damage to the retina has already occurred. Although RGC apoptosis has been identified as the earliest form of cell death in glaucoma, it is estimated that up to 40 % of RGCs are lost before field defects can be detected using the standard clinical tests [94].

The eye offers the unique opportunity to non-invasively image structures of the central nervous system. This review outlines recent developments in our understanding of the underlying mechanisms of glaucoma pathology and explores recent advances towards exploiting these findings to develop novel tools for the early diagnosis of glaucoma, including an overview of recent advances in retinal and brain-imaging technologies. The role of Müller cells and microglia in glaucoma pathology has recently been reviewed elsewhere [161] and, therefore, considered beyond the scope of this article.

## Mechanisms of RGC and axonal death

### RGC loss predominantly occurs by apoptosis

Loss of RGCs has been identified as the earliest form of cell death in glaucoma, and reduction in RGC function is ultimately considered responsible for visual field loss [144]. The human retina contains approximately 1.5 million RGCs [119] with an estimated rate of RGC loss of 0.4 % per year due to normal physiological ageing, increasing to 4 % per year in glaucoma patients [31]. RGC death in glaucoma is thought to predominantly occur through apoptosis, although other forms of cell death do occur [154].

Apoptosis describes a process of controlled cell death, rather than necrosis, which describes disordered and accidental cell death [63]. Apoptosis is a regulated process of cell death leading to chromatin condensation, DNA fragmentation, oxidative damage, and autophagic degeneration, commonly proceeding through either extrinsic or intrinsic caspase-dependent pathways [50]. Due to the complex and multifactorial nature of glaucoma (Table 1), multiple mechanisms are thought to contribute to RGC loss. Observations that axotomy precedes RGC soma loss by a period of several days in rodent models of axotomy and IOP elevation using the retrograde label Fluorogold have long hinted that changes at the level of the RGC axon precede apoptotic degeneration of the RGC soma [15]. More recent support for this view was presented by *Calkins*, who in summarising the results of several rodent studies using the acute IOP Morrison’s model of ocular hypertension found that axonal degeneration typically occurs at a 2–3-fold greater extent than RGC soma loss and exhibits a stronger positive correlation with IOP exposure [18].

Evidence to support the theory of RGC loss as a result of axonal transport failure in glaucoma was first observed in experimental monkey models of IOP elevation by Anderson and Hendrickson, who identified the site of the lamina cribrosa as the primary site of transport dysregulation [3]. Downregulation of retrograde (brain-to-retina) axonal transport in response to anterior chamber cannulation-induced IOP elevation in the rat was reported by Johansson et al. [83]. Experimental data to demonstrate anterograde (retina-to-brain) axonal transport dysregulation was later described in the murine transgenic DBA/2 glaucoma model and microbead occlusion model of raised intraocular pressure [33]. A more comprehensive mini-review outlining the history of axonal transport dysregulation was recently published [51]. Several mechanisms have been proposed to describe the events resulting in axonal transport dysregulation leading to RGC axonal degeneration and soma death, an overview of which is provided in the following sub-sections.

### Mechanical theory of glaucoma progression

The mechanical theory of glaucoma progression suggests that elevated IOP leads to the compression of the nerve fibre bundle, primarily at the site of the Elschnig’s ring and the lamina cribrosa, causing a simultaneous discontinuity of anterograde and retrograde axoplasmic transport [193]. One mechanism by which this was thought to lead to subsequent RGC degeneration and apoptosis induction is via the inhibition of the retrograde transport of Neurotrophic Factors (NFs), such as the Brain Derived Neurotrophic Factor (BDNF) and Neurotrophins 4/5 (NT4/5), from the superior colliculus to RGC soma [84], despite an early compensatory period of increased production of BDNF mRNA by RGCs in optic nerve injury models [62]. Further support for the involvement of neurotrophic factor deprivation in RGC apoptosis was found when exogenous administration of NF proteins, including Nerve Growth Factor (NGF), Ciliary neurotrophic factor (CNTF), and BDNF, protected RGCs from apoptosis in vitro [85] and in vivo models of disease [147]. BDNF stimulates various retinal signalling pathways on the activation of its target TrkB receptor, including the extracellular signal-regulated kinases 1/2 (Erk1/2) and the phosphatidylinositol-3 kinase (PI-3 K)/Akt pathways. Both play a major role in RGC loss in glaucoma [132].

Criticisms of the mechanical theory of glaucoma progression include a lack of correlation between IOP and visual field loss in some patient subsets. For example, racial differences in glaucoma incidence are reported despite similar IOP profiles. In addition, a gender disparity in the prevalence of NTG is observed despite similar IOP profiles [53]. A possible explanation for these observations can be found in consideration of the biomechanical structure of tissues at the site of the sclera and ONH. This paradigm suggests that variations in the biomechanical properties of these tissues may contribute to the variance in susceptibility to IOP-induced deformation of these tissues and glaucomatous visual field loss [43]. Clinical support for ONH and lamina cribrosa deformation in glaucoma patients includes a recent study using spectral-domain optical coherence tomography to visualise the ONH of 173 eyes (from 88 glaucoma patients and 20 normal subjects) at 4 month intervals for a mean duration of 5.3 years [190]. The study reported that both the ONH and anterior lamina cribrosa surfaces were displaced in a significant portion of glaucoma patients and the magnitude of this change was related to patient age and average IOP during follow-up. A recent criticism of this approach, however, is that studies such as these assume ONHs that possess an atypical sensitivity to IOP also have atypical characteristics, which may not necessarily be the case [181]. This suggests that it may be difficult to use eye-specific biomechanical properties as predictors of IOP sensitivity and glaucoma susceptibility in isolation.

Further limitations of the mechanical theory of glaucoma progression were identified when observations from animal studies suggested differences in the rate of inhibition of anterograde and retrograde axonal transport in RGCs. An earlier onset and greater extent of anterograde transport dysregulation was reported in the microbead occlusion and DBA/2 J murine model of glaucoma [33, 39]. A possible solution to the discrepancy in anterograde/retrograde axonal transport dysfunction can be found when consideration is paid to the differences in energy requirement for each of these processes. Anterograde axonal transport is dependent on kinesins which are reported to utilise ATP less efficiently than the retrograde transport protein dynein, which can change its step-size dependent on ATP availability and so better maintain function in an energy scarce environment [116]. The dependence of axonal transport processes on energy availability suggests a role for mitochondrial dysfunction in RGC axonal degeneration inviting an ischemic theory of glaucoma progression.

### Ischemic theory of glaucoma progression

A blood perfusion deficit is the main component of the ischemic theory of glaucoma which states that hemodynamic alterations may act independently from, or in conjunction with, raised IOP to cause damage at the site of the ONH [53]. The ischemic theory of glaucoma has also been suggested to play a fundamental role in the pathogenesis of NTG, with reports of patients with systemic vascular disorders (such as atherosclerotic ischemic stroke) being at significantly greater risk of glaucomatous cupping of the optic disc and bilateral NTG development [98]. In addition, patients with primary vascular dysregulation, which describes a condition associated with sudden vasoconstriction in response to environmental or emotional stimuli in the extremities and the retina, is also associated with increased risk of NTG [54].

Further evidence to support the ischemic theory of glaucoma progression stems from recent developments in non-invasive measurement of ocular blood flow (OBF) which can be achieved with increasing accuracy using functional OCT [171]. A significant reduction in OBF and ocular perfusion pressure in POAG patients is reported versus controls using these techniques [171]. Reduced ocular blood flow is purported to result in RGC loss via induction of a recurrent mild ischemic injury leading to chronic oxidative stress and excitotoxicity [129] or alternatively, by sensitising RGCs to ordinarily well-tolerated secondary insults, such as glutamate, nitric oxide, and phototoxicity [135]. These insults act to impede mitochondrial function, the effects of which are most pronounced at the site of unmyelinated RGC axons at the ONH which, due to the reduced efficiency of signal transduction of unmyelinated axons, have one of the greatest energy demands in the body, manifesting as a high mitochondrial density at this location [103].

The role of mitochondria dysfunction as the cause of RGC axonal transport dysregulation also offers an explanation for the age-dependent increase in glaucoma risk due to reduced mitochondrial efficiency [16] and the increased incidence of genetic mutations associated with mitochondrial dysfunction in POAG patients [10]. Declining mitochondrial function with age is thought to increase susceptibility to additional stressors, such as elevated IOP [33], and has been suggested as a potential mechanism to explain the lack of positive correlation between the clinical correlates of RGC loss and extent of elevated IOP [32]. Mitochondria are highly dynamic organelles which undergo fusion and fission events. They help to mitigate stress by diffusing the contents of partially damaged mitochondria and by facilitating apoptosis during high levels of cellular stress, respectively [198]. Histological observations reveal induction of mitochondrial fission and optic atrophy 1 (OPA1) release in murine models of glaucomatous optic neuropathy [91]. OPA1 regulates mitochondrial fusion and maintenance of cristae structure. Mutations in this protein are responsible for dominant optic atrophy [1], and reduced expression is reported in POAG patients due to increased susceptibility to stress-induced mitochondrial dysfunction [13]. Upregulation of OPA1 expression has been found to protect RGC loss in glaucomatous DBA/2 J mice, suggesting that this protein may provide a therapeutic target for the treatment of POAG [90].

Dysregulation of RGC axonal transport processes is thought to result in RGC death through dysregulation of calcium homeostasis, resulting in increases in cytoplasmic calcium concentrations culminating in increased oxidative stress, calpain-mediated cytoskeletal degradation, and caspase-dependent apoptosis induction [32]. Calcium channel blocker therapies, including nimodipine, brovincamine, and nilvadipine, have been suggested to have beneficial effects on visual function in both animal models and small scale trials in glaucoma patients, however, differentiating neuroprotective and vasodilating effects of these agents has proven difficult with larger multi-centre trials of these agents required [4].

### Autophagy dysregulation in glaucoma

Autophagy describes the orderly self-degradation and recycling of unwanted or dysfunctional cellular components and is used by terminally differentiated cells to manage their cytoskeletal and organelle turnover [101]. Autophagy can be upregulated in response to cellular stress [102] and dysregulation of this process is increasingly associated with neuropathological conditions, including Alzheimer’s and Parkinson’s diseases [134]. In glaucoma models, an apparent upregulation of autophagy (via increased observance of autophagosomes) is reported in RGC soma [38], and dysregulation of autophagic flux is reported in RGC axons in response to downregulation of anterograde axonal transport in the murine DBA/2 J glaucoma model [101].

As a therapeutic target for glaucoma treatment, the role of autophagy in RGC death remains controversial, with both inhibitors (3-methyladenine) and inducers (Rapamycin) reported to elicit neuroprotection in the same rodent glaucoma model [136, 169]. A possible explanation for this discrepancy is that Rapamycin acts on pathways in addition to autophagy modulation such as downregulation of TNF-α [169]. An alternative explanation suggests, while the inhibition of autophagy at the site of the RGC soma can prevent apoptosis induction late in glaucoma progression, inducers of autophagy, such as Rapamycin, may act to upregulate autophagy at the site of the RGC axons and so elicit a neuroprotective action provided that they are administered early in the disease process [130]. Once this controversy is resolved, favourable modulation of  autophagy to preserve RGC function could provide a novel therapeutic target for the treatment of glaucoma.

### Amyloid beta and tau in early glaucoma pathology

Amyloid beta (Aβ) is the major constituent of senile plaques in Alzheimer’s disease (AD), caused by the abnormal processing of Amyloid Precursor Protein (APP). Retinal deposition of Aβ is increasingly thought to contribute to the ocular abnormalities reported in AD patients [146]. Age-dependent accumulation of Aβ in RGCs has been suggested to contribute to glaucoma pathology, and anti-Aβ therapies have been identified as a therapeutic target to prevent RGC degeneration [68]. These data, coupled with reports of an increased incidence of AD among glaucoma patients [113], have led to the suggestion that glaucoma could be described as a type of ocular AD [123].

Aβ production in glaucoma can occur as a result of caspase-3-mediated abnormal APP processing [124], and reduced levels of Aβ_1-42_ and increased levels of Tau are found in the vitreous of glaucoma patients versus age-matched controls, a pattern consistent with that found in the Cerebral Spinal Fluid (CSF) of patients with AD [197]. More recent observations suggest that Aβ_1-42_ is upregulated in the RNFL at the site of the ONH in a monkey model of chronic ocular hypertension [81]. This is of interest as Aβ is well documented to interrupt both intracellular calcium homeostasis [166] and anterograde axonal transport processes [21] in AD, and a similar pathology may occur in glaucomatous RGC degeneration and explain the increased susceptibility of AD patients to glaucoma [12].

Disruption of intracellular calcium homeostasis by Aβ in neurons during AD can occur via a number of processes, such as increasing plasma membrane permeability via the formation of calcium permeable-pores [157]. Aβ-mediated disruption of anterograde axonal transport in hippocampal neurons can occur directly via activation of actin polymerisation by Ras homolog gene family member A (RhoA) [176], or glycogen synthase kinase-3β (GSK-3β) signalling [36].

GSK-3β is an inhibitor of fast anterograde axonal transport implicated in glutamate-induced excitotoxicity [27]. Inhibition of GSK-3β-mediated activation of dynamin-related protein 1 (Drp-1) has recently been demonstrated to reduce Aβ-induced mitochondrial fission and apoptosis in culture and murine models of AD [194]. In the retina, an RNAi screen has recently identified GSK-3β as a regulator of Drp-1, inhibition of which was recently observed to preserve RGC axon integrity in murine glaucoma models [100]. While inhibitors of GSK-3β have been identified as an important strategy for the treatment of CNS disorders including AD [49], to date, there are few studies investigating whether these molecules could be of benefit for the treatment of glaucoma. Elevated levels of RhoA are reported in the ONH of human eyes with glaucoma [66], and Rho kinase (ROCK) inhibitors are increasingly recognised as a promising therapeutic strategy for the treatment of glaucoma, indicating shared pathology [184]. In addition to the well-established IOP-modulating activity of these agents, ROCK inhibitors have been found to promote RGC axonal regeneration in both murine and feline models of optic nerve crush, a subject recently reviewed by Van de Velde et al. [177].

A downstream effect of Aβ-mediated activation of GSK-3β in hippocampal neurons is the tau hyper-phosphorylation, which can stimulate the release of kinesin bound cargos so reducing the efficiency of anterograde axonal transport [92]. A histological study of 19 enucleated human eyes found a significant age-dependent increase in ganglion cell layer Tau protein using a phosphorylation state-independent antibody [105]. A further human study of 11 surgical glaucomatous eyes revealed that Tau is ordinarily present in both the inner nuclear and inner plexiform retinal layers, but concentrations are diminished in glaucoma patients versus age-matched controls [70]. The same study suggests that the concentrations of abnormally phosphorylated (AT8) Tau were elevated in secondary angle closure glaucomatous but not in six POAG eyes, which may imply the extent of Tau hyper-phosphorylation correlates with disease severity. Both Tau and hyper-phosphorylated Tau have been suggested to influence axonal transport processes in cortical neurons via a number of processes [186]. For example, upregulation of Tau is documented to selectively impede anterograde axonal transport of APP and organelles due to the ten-fold higher affinity of Tau for kinesin than dynein [41]. Murine studies have suggested that downregulation of Tau could provide an attractive therapeutic target for the prevention of Aβ-induced dysregulation of axonal transport [182].

### The role of trans-lamina cribrosa pressure difference in glaucoma

An alternative explanation for the variability in response to IOP elevation and extent of glaucomatous RGC damage is that IOP does not wholly describe the pressure situation at the ONH [87]. The change in IOP relative to orbital cerebral spinal fluid pressure (CSFP), the so-called trans-lamina cribrosa pressure difference (TLPD) or a change in the time-dependent relationship between the pulse-synchronous changes in IOP and orbital CSFP, has been suggested to provide a more accurate description of the pressure situation at the ONH [87]. There is growing clinical evidence to support the hypothesis that CSFP and TLPD play an important role in glaucoma pathology. This hypothesis may also explain the increased incidence of glaucoma in patients with a history of migraine (Table 1), as elevated intracranial pressure is associated with this condition [164]. A recent literature review and meta-analysis comprising five papers and a total of 396 patients by Siaudvytyte et al. revealed that CSFP is elevated in glaucoma patients compared to healthy subjects and TLPD is related to changes in optic disc structure in patients with high-tension glaucoma [162]. The authors caution, however, that further experimental investigations and longitudinal prospective clinical studies are required to overcome the methodological weaknesses of the current studies, concerns echoed by Pircher and Killer [138]. Since this time, the Beijing intracranial and intraocular pressure (iCOP) reported a positive association between TLPD (calculated from LP-CSFP) and visual field defects in POAG patients [77] and a population-based study from the Korean National Health and Nutrition Examination Survey (KNHANES) found that TLPD (calculated from estimated CSFP values) was significantly associated with HT-NTG [104].

Further support for a role of CSFP and TLPD in glaucoma pathology is found in animal models. For example, a study by Yang et al. in a monkey model demonstrated optic neuropathy in response to chronic reduction in CSFP despite normal IOP [195]. Further evidence to support this view was recently presented by Hou et al., who found, in canines, that TLPD became uncoupled once CSFP is reduced below a critical threshold [77]. This, coupled with reports that CSFP naturally declines with age, offers an attractive explanation for the efficacy of IOP-modulating therapies for the treatment of NTG and suggests that the management of CSFP in addition to IOP should be considered for a more effective glaucoma treatment strategy [55]. Large-scale clinical application of TLPD measurements is, however, presently limited by the invasive nature of LP-CSFP monitoring and the erroneous assumption that CSFP is uniform between the spinal column and optic nerve subarachnoid space (SAS) [97]. Non-invasive techniques for determining CSFP and TLPD are presently in development [163].

Two mechanisms have been proposed to describe the influence of CSFP and TLPD on glaucoma pathology. The first of which suggests that an abnormal CSFP leads to similar mechanical deformation of the ONH as has previously been reported for elevated IOP [86]. This hypothesis is contested on grounds including that the rigid structure of the lamina cribrosa will resist bowing at TLPDs of clinically relevant magnitude [74]. A second mechanism is that the dysregulation of CSF hydrodynamics, manifesting as elevated CSFP and resulting in accumulation of toxic compounds at the site of the ONH, leads to RGC loss [189]. There has been a recent resurgence in interest in the role of CSF turnover in CNS disorders with the re-discovery of the paravascular pathway for CSF and interstitial fluid exchange in the brain, termed the glymphatic system [82]. Dysregulation of glymphatic mediated clearance of interstitial solutes, including Aβ and Tau, has been suggested as a novel mechanism to explain the increased risk of dementia in patients after traumatic brain injury [80]. The dysregulation of the production and clearance of cytotoxic compounds, such as Aβ, coupled with recent postulations as to the existence of an ocular glymphatic system [40], has led to the development of a novel hypothesis for glaucoma pathology based on the reduction in CSF clearance from the SAS via ocular glymphatic dysregulation [189]. Therapeutic interventions to increase glymphatic clearance such as aquaporins agonists, which are currently under investigation for the treatment of AD, may also be of interest for the treatment of glaucoma [174]. Finally, the recognition that Schlemm’s canal is a VEGF-c responsive vessel with lymphatic phenotype [5], coupled with the observation that the concentration of proteins implicated in cytotoxic processes (inflammation, apoptosis, angiogenesis, and oxidative stress) in the aqueous humour that are elevated in ocular disease [9] may suggest a role for aqueous outflow in the removal of cytotoxic compounds from posterior ocular tissues. This offers an alternative explanation why therapies to increase aqueous outflow may be more advantageous than those which act to reduce aqueous inflow [175].

### The brain in glaucoma

**Fig. 1:**
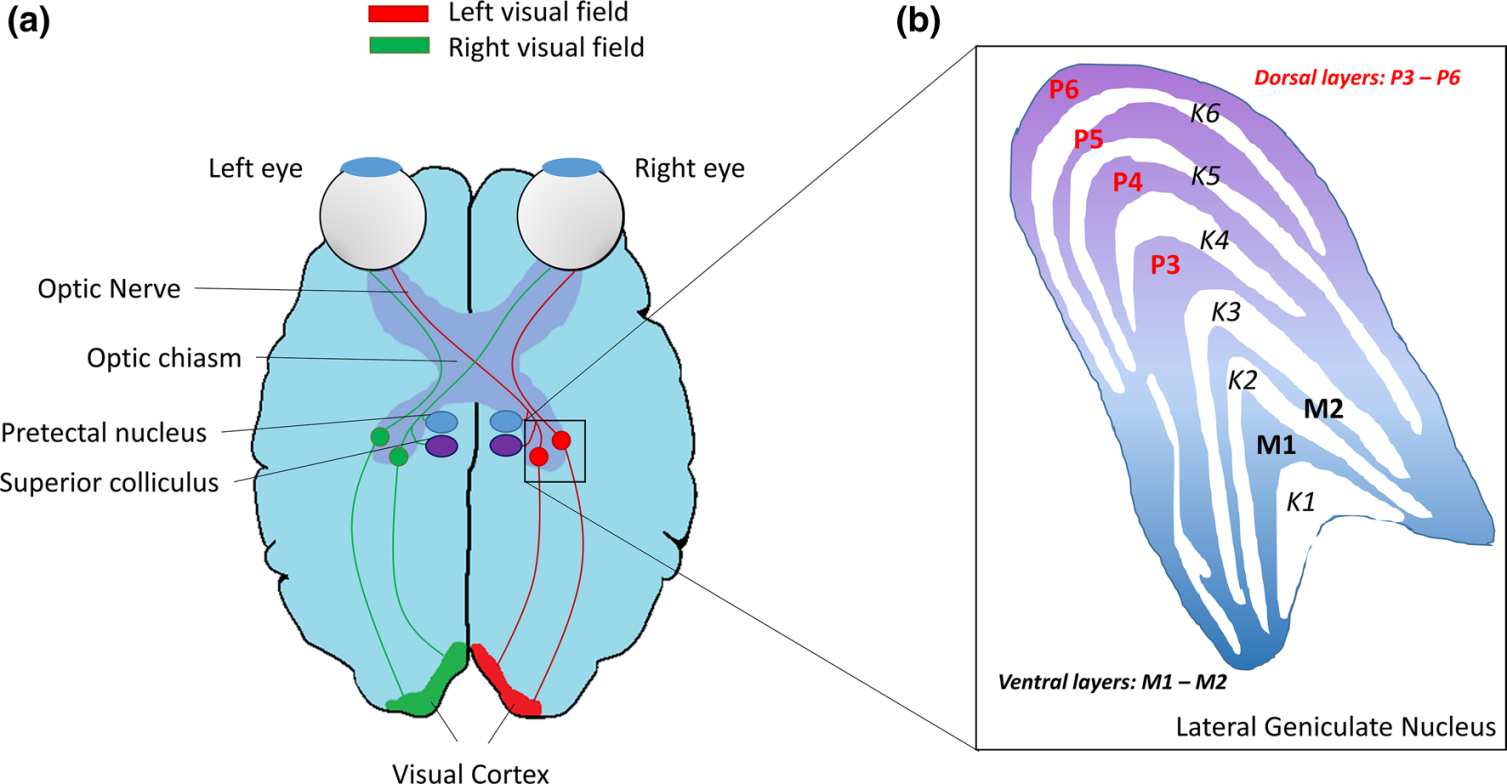
Overview of the human visual pathway. **a** Diagrammatic representation of the human visual pathway. **b** Overview of the structure of the lateral geniculate nucleus illustrating the organisation of magnocellular (M-cells), parvocellular (P-cell), and Koniocellular cells (K-cells)

The observation that glaucoma exhibits pathological similarities with CNS neurodegenerative disorders, coupled with recent advancements in neuroimaging technologies, has led to a growth in interest in the effects of glaucoma on the visual pathway in the brain [72]. The human visual pathway is divided into two parts; the anterior visual pathway is composed of the retina, optic nerve, and chiasm and lateral geniculate nucleus (LGN), while the posterior visual pathway comprises the optic radiations and visual cortex (Fig. 1a).

#### The lateral geniculate nucleus (LGN)

In humans over 90 % of RGC axons project to the LGN, a relay station which in turn projects its axons to the visual cortex via the optic radiation [200]. The remaining 10 % of RGCs project to brain structures, including the superior colliculus (eye-movement control), pretectal area (eye movement/pupillary reflex), accessory optic system nuclei (optokinetic nystagmus), and the suprachiasmatic nucleus (circadian rhythm maintenance) [65]. Both brain hemispheres possess an LGN which primarily receives information from its contralateral visual hemifield and is typically organised into six distinctive layers; with the magnocellular cells (M-cells) in ventral layers (1 and 2) and parvocellular cells (P-cell) occupying the dorsal layers (3–6). Each layer of M- and P-cells is in turn separated by a layer of Koniocellular cells (K-cells), as shown in Fig. 1b [192]. M, P, and K cells of the LGN receive input from their respective M, P, and K RGCs, the M pathway is achromatic and displays high-frequency tuning and the P pathway exhibits chromatic sensitivity and high spatial resolution [2]. K cells are thought to comprise multiple classes which are suggested to contribute short-wavelength chromatic and orientation selectivity [192].

Cells in the LGN respond to visual response fields similar to those found in RGCs. There is approximately a one-to-one mapping of RGC output to LGN output in primates [73], thus there is close matching of receptive field characteristics. Typically, RGC axons from the ipsilateral eye synapse in layers 2, 3, and 5 of the LGN, while axons from contralateral eyes synapse in the first, fourth, and sixth layers [170]. Traditionally, the primary function of the LGN was thought to act as a gateway, relaying visual information from RGCs to the visual cortex [88]. More recent observations that retinal afferents constitute only 10 % of LGN input with additional modulatory inputs from a variety of brain regions (including the striate cortex, the brain stem, and thalamic reticular nucleus), suggests that the LGN instead acts more like a gatekeeper, representing the first stage of the visual pathway, where cortical top-down feedback can influence visual processing [122].

#### The primary visual cortex

The LGN output pathway begins with the projection of thalamocortical axons through the internal capsule to form broadly spreading fibres termed the optic radiations, which terminate in each hemispheres ipsilateral primary visual cortex in the occipital lobe (V1) [140]. As the optic radiations leave the LGN, they are divided into the superior and inferior radiations. Inferior radiations project to inferior areas of the primary visual cortex, providing information regarding the superior visual field, while superior radiations project to superior regions of the primary visual cortex, supplying information on the inferior visual field [140]. LGN relay neurons from the M, P, and K layers remain segregated throughout the optic radiation, projecting to distinct layers of V1 (layers 4Cα, 4Cβ, and 2/3, respectively [24]). In turn, each V1 transmits information via the ventral and dorsal stream to higher order centres in the visual cortical hierarchy (V2–V6). In addition to these feed-forward connections, a series of direct reciprocal and cascading feedback processes enable neurons of the visual system to serve as adaptive processors, adopting different functional roles according to the task being executed [64].

## Techniques for monitoring retinal ganglion cell health

### Standard automated perimetry (SAP)

Standard automated perimetry (SAP) is considered the gold standard for visual field assessment and monitoring of visual field loss, with RGC loss correlating with automated perimetry observations in glaucoma patients [145]. Parameters, such as the mean deviation (MD), pattern standard deviation (PSD), and glaucoma Hemifield test (GHT), have been used for many years. However, these global-indices-based approaches have limitations, which include human error in estimating the rate of progression, the large number of visual field measurements required for trend-based analysis, and the large amount of information lost when compiling SAP data into a single integer of progression [26]. Attempts to overcome these limitations led to the development of event-based and trend-based analysis techniques, such as guided progression analysis (GPA) and visual field index (VFI). GPA involves the comparison of each visual field assessment with a series of baseline tests, with progression defined as a binary outcome (progressor/non-progressor). This technique requires fewer visual field tests (shorter follow-up time) than trend-based analysis techniques but is considered less reliable [75]. Alternative approaches to visual field analysis include the assessment of progression at each individual test location (Progressor) [180], or using structurally relevant test location clusters [8].

Classically glaucomatous visual field loss is described as loss of peripheral vision, however, a prospective evaluation of glaucoma patients’ visual symptoms suggests that early glaucomatous visual defects can be more complex, involving RGC loss in the central retina [78]. Observations such as these have led to a resurgence in interest in the role of central vision defects in glaucoma progression, particularly at the level of the macular [76]. This is problematic for SAP as although macular defects have long been reported in early stage glaucoma [6], the most commonly used SAP test (Humphrey 24-2 visual field test) utilizes points spaced 6° apart [196], which neglects a large portion of the central macula which has an approximate field diameter of 8° [76]. While the macula represents less than 2 % of the total retinal area, it is reported to contain over 30 % of the RGCs [34]. Together, these observations suggest that greater consideration to macular defects should be considered for the diagnosis of glaucoma using SAP.

### Optical coherence tomography (OCT)

Optical coherence tomography (OCT) is a non-invasive optical-imaging technology allowing cross-sectional imaging of the eye in vivo. OCT was first introduced in 1991 and utilizes low-coherence interferometry to generate a two-dimensional cross-sectional image of the retina [79]. The axial and lateral resolutions of OCT are decoupled, with the axial resolution depending on the coherence length of the laser light source and the lateral resolution a function the optical properties of the system, including patient pupil size [202].

Spectral-domain (SD) OCT is capable of scanning speeds up to 110 times faster than its predecessor, time domain (TD)-OCT [160]. SD-OCT employs a broadband light source and a high-speed spectrometer generating a mean imaging range ~3.0 μm in the air, a rapid sensitivity fall-off (~6 dB/1.5 mm), and a 120 kHz scan rate, achieving image capture speeds of approximately 27,000 A-scans/second with typical axial resolution of 6 µm [56]. The high axial resolution enables this technique to reproducibly assess the thickness of retinal structures, including the ability to longitudinally monitor retinal nerve fibre layer (RNFL) changes in clinical glaucoma. The high acquisition speeds of SD-OCT permit the collection of three-dimensional data sets for clinical use. Using this technique, volumetric assessment of structures, including the ONH and macular, can be achieved. To compensate for eye movement during imaging acquisition, OCT images are registered using reference images acquired using confocal scanning laser ophthalmoscope (cSLO) or OCT-mediated blood-vessel alignment [191].

Recent advances in OCT technology have led to the development of so-called functional-OCT technologies [99], including Doppler OCT for the assessment of retinal blood flow and polarisation OCT for the assessment of birefringent tissues, such as ganglion cell axon densities [106]. Improvements to the underlying OCT technology have led to the development of swept source (SS)-OCT [168], Adaptive Optics (AO)-OCT [126], and Frequency-Domain (FD)-OCT [128]. With the resulting enhancements in image resolution through improvements in image acquisition speed and compensation for wavefront aberrations caused by anterior ocular tissues, the resolution of this technique allows visualisation of individual cone photoreceptors cells, RNFL bundles [126], and the thickness of RGC layers [128]. At present, individual RGCs cannot be visualised using OCT due to the near transparent nature of these cells.

### Confocal scanning laser ophthalmoscopy (cSLO)

Confocal scanning laser ophthalmoscopy (cSLO) is presently the most extensively used retinal imaging technique, despite being first described over 35 years ago. cSLO instruments, such as the Heidelberg Retina Tomography III (HRT III; Heidelberg Engineering, Heidelberg, Germany) instrument, incorporate a point laser light source which scans the retina and a confocal pinhole aperture that acts to minimise scattered and reflected light outside the image focal-plane dramatically increasing lateral resolution (10 µm) versus fundus photography and enabling retinal topographic imaging. cSLO has proven to be a useful and versatile tool for the assessment of geographic atrophy [158], epiretinal membrane characteristics [149], drusen deposits [159], fluorescein/indocyanine green angiography [89], RPE [28], retinal nerve fibre layer thickness, and optic nerve head morphology [95]. Despite the wide-range of applications for which cSLO imaging is used, this technique has several well-documented limitations, including impaired imaging in the presence of lens and corneal opacities, small pupils, and limited axial resolution (300 µm) [42]. To address these limitations, several adaptations to cSLO imaging have been developed which are described in the following sub-sections.

#### Adaptive optics

Adaptive optics (AO) presents a technique which can be combined with the conventional cSLO- and OCT-imaging modalities to sufficiently enhance the resolution of cSLO imaging to resolve individual cells, with current practical lateral and axial resolutions of 2.5 and 100 µm, [152] compared to that of conventional cSLO systems which are typically 10 and 300 µm, respectively. Resolution is limited by optical aberrations in ocular tissues and can exhibit a large variation between individual patients and over time [117].

Adaptive optics (AO) was first described by Horace Babcock in 1953 and involves measuring wavefront distortions caused by optical aberrations (such as atmospheric disturbance in ground-based astronomy or corneal and lens effects in retinal imaging) and using a device, such as a deformable mirror or liquid crystal array, to compensate for these distortions [7]. While this technology initially found use for military and astronomical observations, Dreher et al. demonstrated that AO could also be used to enhance the resolution of cSLO retinal imaging in 1989 [44]. Limitations of correcting ocular aberrations using AO include the number of adjustable elements in the deformable mirror and the deflection range of the surface [11]. Today, many AO systems employ microelectromechanical system (MEMS) wavefront correction techniques, which are lower in cost than piezodriven mirrors and spatial light modulators [11].

Since this time AOSLO systems have been successfully used to visualise a variety of ocular tissues, including retinal nerve fibre bundles and photoreceptors at the single-cell resolution [151, 153]. Despite the significant progress in visualising some retinal cell types using AOSLO systems, the label-free visualisation of RGCs using this technique has not yet been achieved. This is because the nerve fibre and ganglion cell layers are approximately 10–20 µm in thickness, which is smaller than the theoretical axial resolution limit of current generation AOSLO systems of 33 µm [150]. Improvement in AOSLO axial resolution to less than 10 µm is required for the label-free imaging of individual RGCs using AOSLO [139]. Suggested improvements to AOSLO systems to reduce axial resolution below the required 10 µm threshold include imaging with longer wavelengths of light which exhibit greater depth of tissue penetration, reducing the size of the confocal pinhole aperture while overcoming associated diffraction artefacts and improving the signal-to-noise ratios through averaging of multiple scans while mitigating eye-movement effects [151].

#### Contrast agents for the in vivo visualisation of healthy RGCs

Despite the many applications of cSLO imaging, the visualisation of RGCs remains extremely difficult due to their almost transparent nature rendering them virtually invisible to optical systems. The development of fluorescent markers of retinal cells has been performed to enhance RGC and cellular contrast for visualisation by cSLO imaging. Due to the central role of RGC loss in glaucoma pathology, it is perhaps not surprising that recent advances in diagnosis of this condition have focused on the development of novel techniques to visualise RGCs. Efforts have included coupling RGC specific contrast agents with cSLO-imaging techniques or the development of novel cSLO-imaging modalities to enhance RGC visualisation in a label-free system. The following sections give an overview of recent developments in each of these fields.

Preclinically, several techniques for labelling healthy RGCs in vivo have been described, including the use of transgenic models expressing fluorescent cell-specific proteins, the utilisation of retrograde fluorescent tracers, and intravitreal administration of contrast agents. Several transgenic models expressing RGC specific fluorescent proteins have been described in rodents, including Thymocyte differentiation antigen 1 (Thy1) [108]. Although transgenic rodent models can achieve labelling efficiencies of ~80 % of total (Fluorogold positive) RGCs, the specificity of labelling using this technique is limited as a result of co-labelling of displaced amacrine cells and microglia due to phagocytosis of fluorescently labelled apoptotic RGCs [185]. Despite these limitations, transgenic models expressing RGC specific fluorescent proteins are increasingly being used to monitor the rate of RGC loss and investigate behaviour of RGC subtypes using established glaucoma models [48].

Retrograde labelling of RGCs involves the application of dyes, such as Fluorogold, carbocyanine dyes (DiAsp, DiI, and DiO), and rhodamine-labelled dextrans typically to the visual centres in the brain, such as the lateral geniculate nucleus [67] or superior colliculus [29], where they undergo retrograde axonal transport to specifically label RGC soma in the retina [173]. Retrograde RGC labelling is a commonly used technique to visualise RGCs in experimental glaucoma models [133], however, due to the invasive nature of dye administration, this form of retrograde RGC labelling is presently unsuitable for clinical translation. Additional criticisms of retrograde labelling of RGCs include incomplete labelling of the RGC population and reduced labelling specificity due to phagocytic uptake of injured but labelled RGCs by microglia and macrophages [185]. Recent attempts to address these criticisms have included the application of retrograde tracers to transected optic nerves, which has resulted in high RGC labelling efficiency (between 96 and 100 %) [155]. In addition, recent observations suggest that the application of the retrograde tracers Fluorogold or Granular Blue directly to the intact rat optic nerve can result in efficient retrograde labelling of RGCs without damaging the optic tract, potentially increasing the likelihood of this technique finding clinical utility [112].

Intravitreal administration of fluorescent markers, such as labelled cholera toxin subunit-B (CTB), has been used to evaluate RGC populations in mice up to 100 days post injection [165]. CTB is an attractive label due to the low cellular toxicity and previous safe systemic administration to patients [156]. Although CTB is not RGC specific, labelling both RGCs and amacrine cells, as the number of amacrine cells is minimally affected in glaucoma models [96] this may not impact the clinical utility of this technique for disease monitoring. The use of intravitreal injection to administer the CTB contrast agent does, however, remain a hurdle to successful clinical translation. Although intravitreal injection of anti-vascular endothelial growth factors (anti-VEGFs) has revolutionised the treatment of common eye disorders, such as neovascular age-related macular degeneration [52], intravitreal injections remain an invasive administration technique unsuitable for the administration of contrast agents in the clinic due to the small but serious risk of side effects [52]. Interestingly, the ability of CTB conjugates to act as retrograde tracers may ultimately permit retrobulbar delivery of this contrast agent into the orbital fat for retrograde labelling of healthy RGCs in patients.

#### Contrast agents for the in vivo visualisation of apoptotic RGCs

**Fig. 2:**
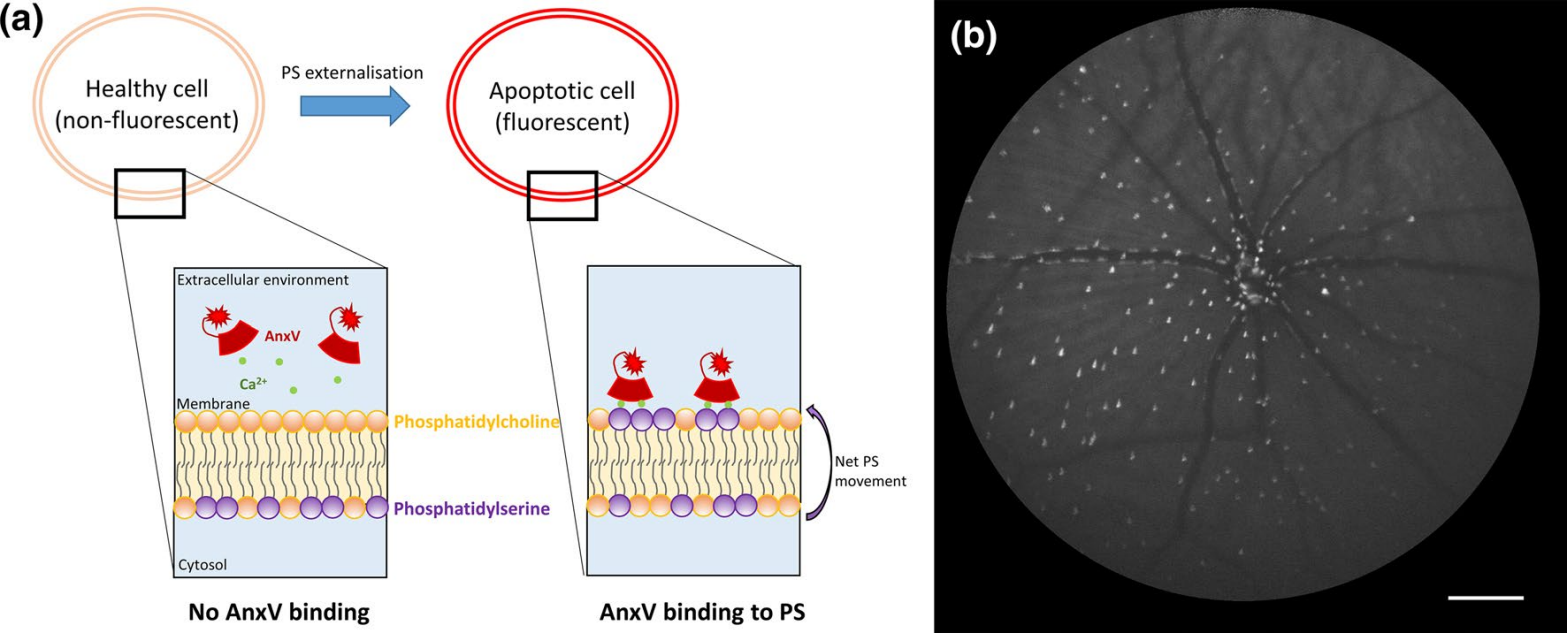
Overview of the DARC technology for the detection of apoptotic retinal cells. **a** Fluorescently labelled annexin A5 (AnxV) preferentially interacts with phosphatidylserine (*purple*) in a calcium-dependent manner. Phosphatidylserine is predominantly localised to the inner leaflet of the plasma membrane of healthy retinal cells via the action of ATP dependent flippases. In apoptotic cells, phosphatidylserine is translocated to the outer membrane leaflet via the downregulation of flippase activity and activation of scramblases, which result in a net increase in phosphatidylserine externalisation. The binding of fluorescently labelled AnxV to this externalised phosphatidylserine permits the visualisation of apoptotic cells at a cellular resolution in vivo. **b** In vivo cSLO retinal image of a Dark Agouti rat with ocular hypertension (Morrison’s model) captured using a 55° lens 2 h after intravitreal administration of fluorescently labelled AnxV (DARC). Apoptotic retinal cells can be clearly visualised as white spots

In addition to cellular labelling, a major advance has been the in vivo visualisation of cellular processes. Apoptosis imaging has been advocated by our group and others using annexin A5 and caspase markers. Annexin A5 electrostatically interacts with negatively charged lipids, including phosphatidylserine, in the presence of divalent cations such as calcium [125]. This is significant as in heathy cells phosphatidylserine is predominantly localized to the inner leaflet of the plasma membrane, however, during apoptosis downregulation of flippase activity coupled with activation of scramblases collectively results in the exposure of phosphatidylserine on extracellular membrane leaflet of the apoptotic mammalian cell (Fig. 2) [109]. Fluorescently conjugated annexin A5 is a well-established tool for the detection of apoptotic cells in vitro (i.e. FITC-conjugated Annexin V [137]). More recently, Technetium-99 m (99 m Tc) radiolabelled annexin A5 has been used in vivo to detect apoptosis in over 30 clinical trials for a range of disorders, including acute cardiac allograft rejection, acute myocardial infarction, ischemic brain injury, cerebral hypoxia, breast cancer, lung cancer, lymphoma, sarcoma, and hepatitis [148]. Externalisation of phosphatidylserine is also reported in cells undergoing necroptosis, which can also be identified using fluorescently conjugated annexin A5 [137].

Our group has recently developed a technology called the detection of apoptotic retinal cells (DARC). DARC uses a fluorescent dye covalently conjugated to the protein annexin A5 in a 1:1 stoichiometry. It was first used to visualise apoptotic RGCs in experimental glaucoma in vivo using non-invasive wide-angle cSLO imaging [30]. Since this time, DARC has been successfully applied to different experimental disease models and has been used to evaluate the neuroprotective effects of drug candidates and monitor the efficacy of therapeutic intervention [61]. A Phase I clinical trial of intravenously administered DARC (ISRCTN59484478) in healthy and glaucoma subjects has recently been completed with results to be published shortly.

TCapQ technology comprises a cell-penetrating Tat-peptide conjugated to an effector caspase recognition sequence (DEVD), a quencher (QSY7), and a fluorophore (Alexa Fluor- 488) which is activated by caspase 3 and 7 in the cytoplasm of pro-apoptotic cells [17]. In an NMDA-induced rat model of glaucoma, intravitreally administered TCapQ was found to enable visualisation of apoptotic RGCs using cSLO, a result confirmed by ex vivo TUNEL staining [143]. Since this time, an improved cell-penetrating caspase-activatable probe, KcapQ, has been developed with reduced toxicity [121]. KcapQ incorporates an improved cell-penetrating peptide sequence (KKKRKV), is reported to exhibit a greater sensitivity to effector caspases, and a greater quenching efficiency between the fluorophore-quencher pair than TcapQ [121]. This technology, however, remains in the preclinical development stage.

### Assessment of glaucomatous change in the brain using MRI

Having previously considered the role of RGCs in glaucoma progression, this section of the review will evaluate the evidence for the involvement of the LGN and visual cortex in glaucomatous degeneration.

Studies of glaucomatous degeneration in the brain were originally derived from experimental monkey models of glaucoma, which identified trans-synaptic degeneration at the site of the LGN and V1 in response to increased IOP and optic nerve damage [201]. Histological observations from monkey models were supported by those from post-mortem human studies that reported a significant reduction in M-cell density in five glaucoma patients compared to controls [25]. A subsequent post-mortem clinicopathological case-report described degeneration of the intracranial optic nerve, LGN and visual cortex in a 79 year old glaucoma patient [69]. Since this time, the advent of non-invasive brain-imaging techniques, such as Magnetic Resonance Imaging (MRI), has led to a rapid growth in studies investigating the involvement of the visual pathway in glaucoma pathology. Structural MRI studies have revealed a significantly shorter optic chasm height and reduced optic nerve diameter in glaucoma patients versus normal controls [203]. Neurodegenerative changes in the LGN, as evidenced by reduction in LGN height and volume, is also reported in POAG patients using voxel-based morphometry and diffusion tensor imaging, with some evidence emerging to suggest that the extent of atrophy may correlate with disease severity [183]. A reduced LGN volume is also reported in patients with NTG versus age-matched controls, which may indicate a shared pathology in the brain [203].

In addition to LGN atrophy, a reduction in volume of the optic radiations and primary visual cortex is documented in patients with POAG [183]. There is some evidence to suggest that a loss of grey matter density occurs in the calcarine sulcus and primary visual cortex in response to prolonged sensory deprivation (AMD and glaucoma patients versus age-matched controls) [14]. More recent studies have reported inconsistencies in grey matter density and glaucoma pathology [110, 188]. This is suggested to occur due to differences in the disease progression between POAG patients included in these studies [110], with an early increase in the grey matter density reported to precede a decline later in the disease process [188]. The early increase in grey mater may be a result of inflammatory processes, such as microglia activation, which is reported in the human glaucomatous ONH [199] and throughout the anterior visual pathway in rodent glaucoma models [45].

Manganese (II) chloride has been employed as a contrast agent for diffusion tensor MRI imaging owing to Mn^2+^ ions being paramagnetic and acting to shorten the spin lattice relaxation time constant of tissues in which it accumulates [118]. Mn^2+^ contrast agents are particularly useful for visualisation of the visual pathway due to its ability to act as a calcium analogue and enter neurons via voltage gated Ca^2+^ channels. Once in the cytoplasm, Mn^2+^ ions are shuttled along axons by microtubual dependent axonal transport processes and can cross synaptic junctions to neighbouring neurons [118]. To date, Mn^2+^ contrast MRI has been utilised to demonstrate significant anatomical age-related differences in rodent models of glaucoma and healthy controls, including an increase in ocular perimeter (15 vs. 3 %), an 8 % reduction in retinal layer thickness, and a negative correlation between optic nerve head width with axonal density (*r* = 0.62, *P* = 0.02) [20]. More recently, this technique has been employed for retinotopic mapping of the superior colliculus in Sprague–Dawley rats after partial optic nerve transection [23]. The authors demonstrate that reduced anterograde axonal transport can be effectively monitored to the sub-millimeter resolution using Mn^2+^ contrast MRI; however, the narrow range between diagnostic and toxic doses of Manganese (II) chloride will require the development of better tolerated alternatives (Mn-chelates) for the translation of this technology to the clinic.

Findings from functional MRI (fMRI) investigations also suggest that changes in the primary visual cortex correlate with anterior visual pathway disease severity [167]. In POAG patients, Quing et al. (2010) and Gerente et al. (2015) used blood oxygen-dependent fMRI to demonstrate that POAG may lead to reduced cortical activity in the primary visual cortex, which correlates with disease severity [142]. Finally, recent resting-state functional MRI studies report alternations in the connectivity of the primary visual cortex and in connectivity between the primary and higher visual centres in POAG patients in response to RGC loss [35]. Care should be taken, however, when interpreting the results of fMRI studies given the extremely high false-positive rate recently associated with this technique [47].

#### Clinical relevance of glaucomatous brain changes

**Fig. 3:**
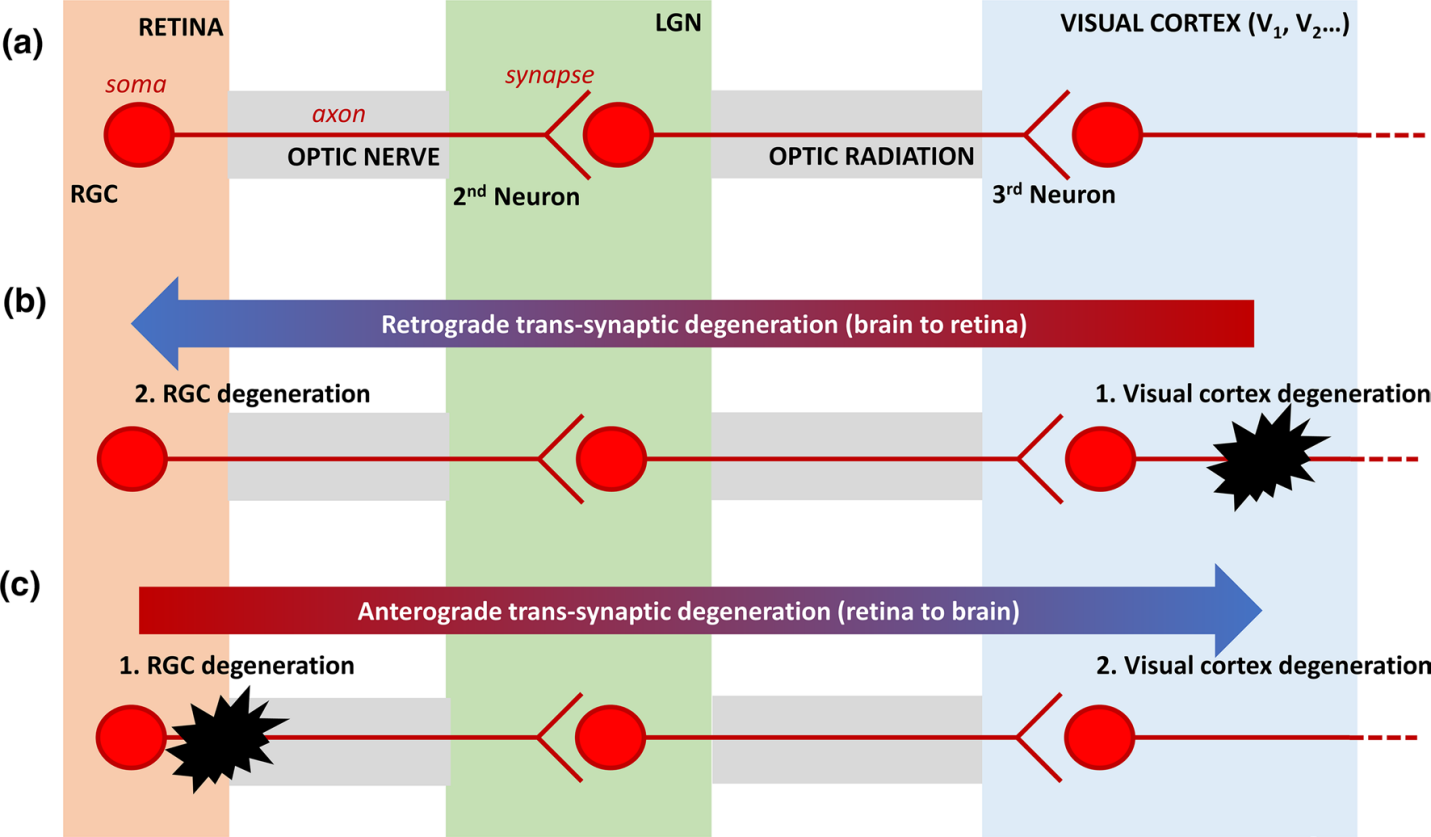
Anatomical depiction of retrograde and anterograde trans-synaptic degeneration in the visual pathway. **a** Diagrammatic representation of the organisation of the anterior (retina, optic nerve, chiasm, optic tracts and LGN) and posterior (optic radiations and visual cortex) visual pathway. **b** Retrograde trans-synaptic degeneration describes the process through which damage to the posterior visual pathway (*black*) results in subsequent retinal degeneration. **c** Anterograde trans-synaptic degeneration describes a process where retinal degeneration leads to a subsequent degeneration of the posterior visual pathway

The observation that brain changes occur in glaucoma patients begs the question as to whether these changes precede or follow the well-established neurodegeneration of RGCs and their axons in the anterior visual pathway. Although the timeline of the natural history of brain changes in glaucoma is at present poorly described, observations of retrograde trans-synaptic degeneration in a variety of neurodegenerative diseases (including Alzheimer’s disease [114] and Multiple Sclerosis [59]), coupled with similarities in the pathology between glaucoma and Alzheimer’s disease may suggest retrograde trans-synaptic degeneration (Fig. 3) is responsible for RGC loss in glaucoma [141]. Some additional evidence to support this view can be derived from monkey models of ocular hypertension, which report that depravation of visual input via RGC loss is not solely responsible for degeneration of the visual pathway [71]. The prevailing view currently, however, is that the majority of glaucomatous degeneration of the posterior visual pathway is preceded by the degeneration of RGC axonopathy, implying anterograde trans-synaptic degeneration is responsible for glaucomatous brain changes (Fig. 3) [19].

While the high cost and limited accessibility to MRI facilities compared to ophthalmic examinations are likely to limit the utility of brain imaging as a front-line glaucoma diagnostic or monitoring tool as suggested elsewhere [111], the identification of neurodegeneration in the visual centres of the brain has important implications for the treatment of glaucoma. Emerging therapeutic interventions intended to restore vision in glaucoma patients, such as RGC transplantation [178] and artificial retinal implants [120], will need to consider the condition of the posterior visual pathway as a confounding factor in the efficacy of therapeutic interventions, including the extent of regeneration of the brain’s visual centres in response to reanimation of the anterior visual pathway [141]. The observation of glaucomatous neurodegeneration in the brain also has implications for assessing the efficacy of neuroprotective therapies, suggesting that the focus of these therapies should be extended to include the whole CNS and that the visual centres of the brain should be included as endpoints in studies of therapeutic efficacy in glaucoma trials [141].

To that end, Weber et al. [187] recently reported that supplementing intravitreal BDNF therapy with injection directly to the visual cortex resulted in a significant enhancement in RGC survival in a feline optic nerve crush injury model versus intravitreal administration of BDNF alone. However, Dekeyster et al. [37] found that viral-vector-mediated upregulation of BDNF in the superior colliculus alone was unable to elicit a significant neuroprotective activity in murine models of optic nerve crush or laser-induced ocular hypertension. The authors suggest their observations to be a result of viral-vector-induced neurotrophin desensitisation, resulting in a failure of BDNF expressed in the superior colliculus to reach the retina via retrograde transport in both healthy mice and injury models. A possible explanation for this is that viral-induced expression of BDNF acts to reduce TrkB-FL expression, a prerequisite for vesicular retrograde transport of this protein. In addition to its neuroprotective activity, however, overexpression of BDNF is increasingly recognised to increase susceptibility to excitotoxicity [131] and downregulation of TrkB-FL is reported in response to excitotoxicity [179]. Viral-induced over production of BDNF may promote excitotoxicity vulnerability, precluding the neuroprotective effects of this agent. This finding has important implications for the growing range of disorders for which recombinant BDNF therapy is being investigated [115].

Finally, upregulation of BDNF secretion and TrkB-activation has been suggested as a potential mechanism underlying the ability of transcranial direct current stimulation to enhance neuronal function in mice [58]. Alternating current stimulation was recently demonstrated in an interventional randomised clinical trial to result in a significant mean improvement in visual field versus sham-treatment controls [24.0 % (*n* = 45) vs. 2.5 % (*n* = 37)] in patients with optic neuropathy, which persisted at 2 month follow-up [60]. Together, these studies provide tantalising evidence to suggest that future glaucoma therapies lie beyond the retina as the primary site of action, instead targeting the whole visual pathway.

## Summary

A better understanding of the mechanisms underlying RGC loss is essential to the development of novel therapeutic interventions to slow or prevent glaucomatous degeneration. The growing recognition that glaucoma is a disease of the whole visual pathway and not just the retina has led to a recent expansion in the number and variety of techniques with the potential to permit the diagnosis of disease before onset of visual field defects. The difficulty of direct visualisation of RGCs in the retina has limited the early diagnosis of glaucoma, as considerable loss of RGCs occurs before disease symptoms manifest. The expense of brain-imaging technologies, recent controversies regarding the validity of fMRI investigations, and suggestion that brain changes occur downstream of those in the optic nerve and retina suggest that while these technologies are likely to provide interesting mechanistic insights into the disease process, they are unlikely to be adopted for the purpose of early diagnosis or screening of disease in the population. Progress towards the clinical translation of RGC specific contrast agents, including the DARC technology, therefore provides the most likely early diagnostic screening tool for glaucoma in the clinic. Finally, the increasingly recognised overlap in the mechanisms of neuronal loss in neurodegenerative disease suggests that a better understanding of glaucoma could inform the development of novel treatment paradigms for other neurodegenerative disorders (and vice versa), with recent studies providing tantalising evidence to suggest that the future of glaucoma therapies lie beyond the retina as the primary site of action, instead targeting the whole visual pathway.
